# *Nostoc* sp. extract induces oxidative stress-mediated root cell destruction in *Mimosa pigra* L.

**DOI:** 10.1186/s40529-014-0081-3

**Published:** 2015-02-22

**Authors:** Siriphan Sukkhaeng, Nuttha Sanevas, Srisom Suwanwong

**Affiliations:** Department of Botany, Faculty of Science, Kasetsart University, Bangkok, 10900 Thailand

**Keywords:** Nostoc sp, Oxidative stress, Lipid peroxidation, Cell death, Root ultrastructure, Mimosa pigra

## Abstract

**Background:**

*Mimosa pigra* is an invasive weed in some regions of South East Asia and Australia. Our previous study has revealed that a cyanobacterium, *Nostoc* sp., extract can inhibit root growth in *M. pigra* seedlings. In this study, some physiological processes involve oxidative stress-mediated cell death and root ultrastructure were investigated to clarify the mechanisms of root growth suppression and bioherbicidal potential of the extract.

**Results:**

*Nostoc* sp. extract enhanced overproduction of reactive oxygen species (ROS) at 24 h, the intensity of red fluorescence increased at 72 h, and caused a slightly increased H_2_O_2_ consistent with the activation of scavenging enzymes (catalase, ascorbic acid peroxidase, glutathione reductase, and peroxidases). This suggests that oxidative stress occurred in the presence of the extract which was supported by increased cell death and lipid peroxidation at 24 h. Reduction of malondialdehyde content and an increase in cell death at 72 h indicated oxidative damage and cellular leakage. Ultrastructural changes were determined at 72 h by scanning electron micrographs which confirmed the damage of epidermal and root cap cells and the disaggregation and destruction of root tip cells. Transmission electron micrographs showed the dissolution of the middle lamella, deposition of some substances in vacuoles, and abnormal mitochondria (swollen mitochondria and indistinct cristae).

**Conclusions:**

*Nostoc* sp. extract enhance oxidative stress by ROS production resulting in lipid peroxidation and massive cell death despite the activation of antioxidative enzymes. Understanding mechanism of action of *Nostoc* sp. extract will provide information for application of the extract to use as natural herbicide for control of *M. pigra*.

**Electronic supplementary material:**

The online version of this article (doi:10.1186/s40529-014-0081-3) contains supplementary material, which is available to authorized users.

## Background

Cyanobacteria are known to produce various kinds of secondary metabolites that can affect many biochemical processes within cells and can influence the growth of surrounding organisms (Leflaive and Ten-Hage [2007]). Natural products from cyanobacteria exhibit various biological inhibitory effects which are cytotoxic, antibacterial, antifungal, and antialgal (Etchegaray et al. [2004]; Kreitlow et al. [1999]; Kulik [1995]). Cyanobacteria have been recognized as options for novel bioactive natural products and use as bio-control agents and herbicides due to their inhibitory activities against some aquatic and terrestrial plants, especially weeds (Duke et al. [2002]; Berry et al. [2008]).

*Nostoc* sp., a filamentous cyanobacterium (Nostocales), has been reported to produce some chemical substances affecting on various organisms. For example, an alkaloid nostocarboline had an inhibitory activity on *Microcystis aeruginosa* (Blom et al. [2006]). Nostocyclamide, a cyclic peptide, had antialgal activity and growth inhibitory activities against cyanobacteria and Chlorophyceae (Kobayashi and Kajiyama [1998]). The effect on higher plants was reported by Hirata et al. ([2003]) that nostocine A produced by *Nostoc spongiaeforme* TISTR 8169 exhibited an inhibitory activity on root growth of barnyard grass (*Echinochloa crus*-*galli* (L.) P. Beauv.). It is possible that *Nostoc* species has weed suppressing potential. Not much is known about the mechanism of *Nostoc* extract.

Understanding the mechanism of natural plant compounds is important for research in natural herbicides. Under unfavorable conditions, such as high or low temperature, water deficit, salinity, and some chemical substances, normal metabolisms of plants are disturbed and toxic molecules are produced (Mano [2002]). One of these toxic molecules is ROS which can abstract hydrogen atoms from polyunsaturated fatty acids (the composition of biological membranes), initiate lipid peroxidation, and cause cell death. During this processes, plants suffer from oxidative stress and malondialdehyde (MDA) is produced as a by-product (Halliwell and Chirico [1993]; Gutteridge [1995]). Because of ROS toxicity, plant cells have mechanisms to reduce these toxic compounds by ROS scavenging via antioxidative enzyme systems (Mittler [2002]). Root ultrastructural changes have been used to study the damage of structures and organelles in root tips exposed to some allelochemicals. These include the decreases in the number of ribosome, dictyosome, mitochondria, endoplasmic reticulum, and metabolic products, mitochondrial swelling and loss of cristae, dissolution of the middle lamella, and the irregular-shaped cells (Burgos et al. [2004]; Jiang and Liu [2010]; Yang et al. [2011]).

In our previous study, crude extract from *Nostoc* sp. was effective on shoot and root growth suppression of *M. pigra* and had an adverse effect on mitotic cell division (Sukkhaeng et al. [2014]), but the inhibition of cell division might not be the only major mechanism of root growth suppression. The aims of this study were to explore the effect of *Nostoc* sp. extract on ROS-mediated oxidative stress (superoxide anion radical (O_2_•^−^) and lipid peroxidation), ROS metabolism in terms of the alterations of antioxidative enzymes, and oxidative damage (cell death and root ultrastructural changes).

## Methods

### Cyanobacteria culture and extraction

A cyanobacterium, *Nostoc* sp. (BotKU C3004, voucher specimen is deposited at herbarium of the Department of Botany, Faculty of Science, Kasetsart University) was collected from rice straw in Bangkok, Thailand and isolated by the streak plate method. *Nostoc* cells were cultured in BG-11 liquid medium (Rippka et al. [1979]), pH 6.5 under daylight fluorescent lamps (300 μmol m^−2^ s^−1^) at ambient temperature (32 ± 3°C). The cells in the exponential phase of growth (15 days after culture) were harvested by means of filtration and subsequently dried at 50°C for 72 h. The dried cells were ground to powder and extracted with 80% methanol for 24 h at ambient temperature. The solution was filtered and the supernatant was evaporated in a rotary evaporator to obtain a crude brown gum. The residue was extracted twice and the supernatant was combined with the first one.

### Bioassay

The gummy substance was dissolved in water containing 0.1% dimethyl sulfoxide (DMSO) at 0, 0.1, 0.3 and 0.5%. Test solutions of various concentrations (1.5 ml) were added to petri dishes (5 cm in diameter) containing filter paper. Six seeds of *M. pigra* were placed on the filter paper and kept in the dark at ambient temperature for 24 and 72 h. The water containing 0.1% DMSO was used as a control.

### Determination of antioxidative enzyme activities

Three hundred milligrams of frozen roots were crushed to a fine powder in a mortar under liquid nitrogen and homogenize with 3 ml of 25 mM potassium phosphate buffer (pH 7.8) containing 0.4 mM EDTA-4H, 1 mM ascorbic acid, and 2% PVPP. The homogenate was centrifuged at 12,000 rpm for 20 min at 4°C and the supernatant was used as an enzyme extract for the assays of catalase (CAT; EC 1.11.1.6), ascorbic acid peroxidase (APX; EC 1.11.1.11), glutathione reductase (GR; EC 1.6.4.2), and peroxidases (PODs; EC 1.11.1.7) activities.

CAT activity was assayed by determination of the initial rate of hydrogen peroxide disappearance according to Aebi ([1983]). Activity was measured spectrophotometrically at 240 nm (E = 0.0394 mM^−1^ cm^−1^). GR activity was determined by the method of Halliwell and Foyer ([1978]). Activity was determined at 340 nm by measuring the decrease of NADPH absorbance (E = 6.1 mM^−1^ cm^−1^). APX activity was assayed by the method of Nakano and Asada ([1987]). Activity was determined at 290 nm by measuring the decrease of ascorbic acid (E = 2.8 mM^−1^ cm^−1^). PODs activity was determined by the method of Putter ([1974]). PODs activity was measured spectrophotometrically at 436 nm by the formation of guaiacol dehydrogenation products (E = 25.5 μM^−1^ cm^−1^).

### Histochemical detection of ROS

ROS production was estimated using a superoxide anion radical-specific oxidation-sensitive fluorescence dye, dihydroethidium (DHE) (Sunohara and Matsumoto [2008]) with a minor modification. Treated roots were stained with 10 μM DHE in 100 μM CaCl_2_, pH 4.75, by gently shaking for 2 h at 25°C in the dark. The roots were then soaked in 100 μM CaCl_2_ for 5 min and observed using a fluorescence microscope (Nikon E600 with a B-2A filter combination, excitation 450–490 nm, emission ≥ 520 nm).

### H_2_O_2_ determination

Hydrogen peroxide content was determined according to Velikova et al. ([2000]). This method is based on potassium iodide (KI) oxidation by H_2_O_2_ to give iodide ion. Iodide reacts with iodine to form triiodide (I_3_^−^) which show absorption at 285 nm (Junglee et al. [2014]). For every sample, distilled water was used instead of KI for tissue coloration background. H_2_O_2_ standard solutions were prepared for calibration curve.

### Determination of root cell death

Treated roots were stained with Evans blue solution (0.025% (w/v) in 100 μM CaCl_2_, pH 5.6) for 30 min. Stained roots were washed 3 times with 100 μM CaCl_2_ (pH 5.6), until the dye no longer eluted from the roots (Yamamoto et al. [2001]). Ten root tips were excised (3 mm) and allowed to soak in 200 μl of *N*,*N*-dimethylformamide for 24 h. The absorbance of released Evans blue was measured at 600 nm.

### Determination of lipid peroxidation

The thiobarbituric acid test, which determines MDA as an end product of lipid peroxidation (Velikova et al. [2000]), was used. The amount of thiobarbituric acid-reactive substances, red pigments, was calculated from the extinction coefficient 155 mM^−1^ cm^−1^.

### Root ultrastructural studies

For scanning electron microscopy, excised root tips of 5 mm (0 and 0.5% at 72 h treated roots) were prefixed in 2.5% glutaraldehyde in a 0.1 M sodium phosphate buffer (pH 7.2), postfixed in 2% osmium tetroxide for 2 h., dehydrated in an acetone series, and dried in critical point dryer (Emitech; K850). After sputter-coating with gold by using an ion coater (Eiko Engineer; IB-2), they were examined with a scanning electron microscope (SEM) (Jeol; JSM5600LV) which operated at 10 kV. For transmission electron microscopy, 3 mm root tip segments were fixed overnight in 2.5% glutaraldehyde in a 0.1 M sodium phosphate buffer (pH 7.2). They were postfixed for 2 h in 2% osmium tetroxide in water. The roots were embedded in a Spurr’s resin and polymerized for 7 h at 80°C. Next, 60 nm cross-sliced sections, probably located in the middle part between the root tip and the quiescent center in the root cap, were made on an ultramicrotome (Leica; UCT) and stained with 5% (w/v) uranyl acetate and 2% (w/v) lead citrate. After fixing, the specimens were examined with a transmission electron microscope (TEM) (Jeol; JEM1220) which operated at 80 kV.

### Statistical analysis

All statistical analyses were performed as analysis of variance (ANOVA) and means were compared using Duncan’s multiple range test (DMRT) at *p* ≤ 0.05.

## Results and discussion

**Figure 1:**
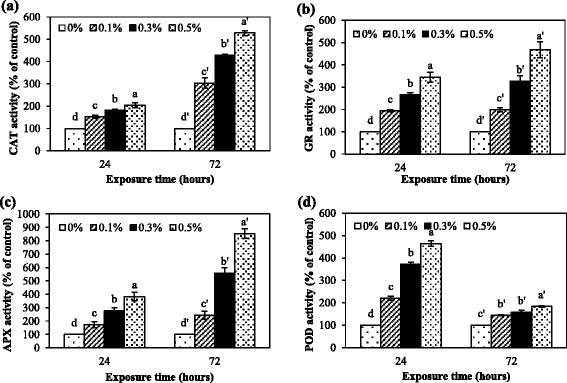
**Antioxidative enzyme activities in** ***M*** **.** ***pigra*** **roots after exposure to the extract at different concentrations for 24 and 72 h.** CAT **(a)**, GR **(b)**, APX **(c)**, and PODs **(d)** Data are mean values of three replications and SD are indicated by vertical bars. The different letters above the column indicate significant differences (*p* ≤ 0.05) by DMRT.

Responses of antioxidative enzyme activities to *Nostoc* sp. extract were determined as an indicator of oxidative stress. Antioxidative enzymes are involved in the detoxification and balance of reactive oxygen species (ROS) when oxidative stress occurs (Mittler [2002]). After 24 h of exposure, CAT, APX, GR, and PODs activities significantly increased with increasing extract concentration (Figure 1a-d). CAT and PODs are enzymes which convert H_2_O_2_ to water and protect the cells from the damaging effects of H_2_O_2_ − a precursor of hydroxyl radical (HO^•^) which is a high oxidative power molecule. APX and GR are enzymes which are involved in the ascorbate-glutathione cycle. CAT and the ascorbate-glutathione cycle are important in H_2_O_2_ scavenging. CAT has a high reaction rate, but a low affinity for H_2_O_2_, so its function is only removing the bulk of H_2_O_2_. APX has a higher affinity for H_2_O_2_ so it can scavenge small amounts of H_2_O_2_ in more specific locations (Dat et al. [2000]). For PODs activity that highly increased at 24 h after treatment but drastically reduced at 72 h indicating a sensitivity of PODs at the initial stress condition (24 h) but not after prolonged stress (72 h). This was similar to the response of guaiacol peroxidases to cadmium that was decreased with high concentration but not with low concentration (Cho and Seo [2005]). Other enzymes involved in H_2_O_2_ scavenging (APX and CAT) seemed to be principal than PODs at 72 h after treatment. Several studies have been reported that there is an increase in antioxidative enzyme activities upon allelochemical stress; for example, Pflugmacher et al. ([2006]) reported that enhanced activities of SOD, CAT, PODs, and GR in alfalfa (*Medicago sativa* L.) in response to oxidative stress caused by the exposure to cyanobacterial metabolites. Essential oils from redstem wormwood (*Artemisia scoparia* Waldst & Kit.) induced oxidative stress in wheat (*Triticum aestivum* L.) roots and elevated antioxidative enzyme activities (Kaur et al. [2012]). All antioxidative enzyme activities kept increasing within 72 h, except for PODs (Figure 1a-d). These results suggest that *Nostoc* sp. extract might cause oxidative stress resulting in a significant activation of antioxidative enzyme activities.

**Figure 2:**
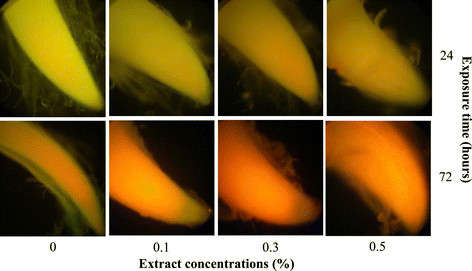
**ROS production in** ***M*** **.** ***pigra*** **roots after exposure to the extract at different concentrations for 24 and 72 h (upper and lower rows).** Red fluorescence shows ROS production (presumably O_2_•^−^).

Effect of *Nostoc* sp. extract on ROS (superoxide anion radicals (O_2_•^−^) and H_2_O_2_) production were detected. Dihydroethidium has been widely used to evaluate ROS production, mainly of O_2_•^−^. It can freely permeate cell membranes and react with O_2_•^−^ to form a red fluorescent product (Gomes et al. [2005]). Our results showed a slightly red fluorescence at 0.3 and 0.5% of the extract concentrations after exposure for 24 h. After 72 h, roots were stained stronger and produced a brighter fluorescence at a concentration 0.1-0.5% than in the control (Figure 2). This indicated that the extract induced ROS production (presumably O_2_•^−^), but the exact mechanism is not known. There were reports that ROS can be generated as by-products of biotransformation reactions of toxins (Lee and Farrell [2001]; Pflugmacher [2004]; Lushchak [2012]). Superoxide anion radicals can be produced from mitochondria when respiration is suppressed, NADPH oxidase on plasma membranes, peroxisome, and endoplasmic reticulum and serves as the precursor of other harmful ROS (Sharma et al. [2012]).

**Figure 3:**
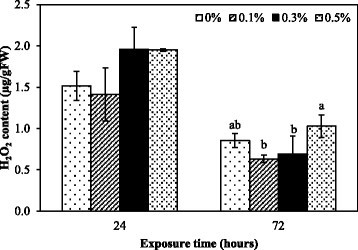
**Hydrogen peroxide content in** ***M*** **.** ***pigra*** **roots after exposure to the extract at different concentrations for 24 and 72 h.** Data are mean values of three replications and SD are indicated by vertical bars. The different letters above the column indicate significant differences (*p* ≤ 0.05) by DMRT.

H_2_O_2_ content in treated roots was not drastically different from the control at 24 and 72 h after treatment but the trends of the H_2_O_2_ content were increased at both times of exposure (Figure 3). H_2_O_2_ is one of ROS molecule which can be converted from O_2_•^−^ and these two molecules were used to indicate oxidative stress level in many researches (Bai et al. [2009]; Lara-nuñez et al. [2006]). The results showed that the treated roots induced the higher production of O_2_•^−^ and H_2_O_2_ than in the control confirming the potential of *Nostoc* sp. extract in oxidative stress induction. However, ROS also correlated with antioxidative enzyme activities. The results of antioxidative enzyme activities except for PODs showed the increase at 24 and 72 h indicating the occurrence of oxidative stress. The cells have to induce the antioxidative enzyme activities to balance ROS level resulting in slight difference of H_2_O_2_ content between treatment and control. This was supported by the study of Singh et al. ([2007]) who found that arsenic induced oxidative stress in mung bean (*Phaseolus aureus* Roxb.) which was corresponded to the increase of antioxidative enzyme activities, but not to H_2_O_2_ accumulation.

**Figure 4:**
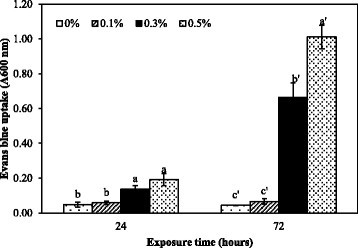
**Uptake of Evans blue in** ***M*** **.** ***pigra*** **root tips after exposure to the extract at different concentrations for 24 and 72 h.** Data are mean values of three replications and SD are indicated by vertical bars. The different letters above the column indicate significant differences (*p* ≤ 0.05) by DMRT.

Cell death occurred after roots were exposed to the extract for 24 and 72 h. This was indicated by relative Evans blue uptake (Figure 4). At 24 h, the uptake of Evans blue was not different from the control with roots exposed to the extract at a concentration of 0.1%, but the uptake gradually increased at 0.3 to 0.5%. Evans blue uptake of treatment with 0.3 and 0.5% dramatically increased about 7 and 11 times more than that of the control at 72 h, respectively (Figure 4). Evans blue is a non-permeating stain which can pass through a damaged plasma membrane of a dead cell and is generally used to indicate the degradation of the plasma membrane and cell death (Peterson et al. [2008]). Our results indicated that the extract caused the loss of membrane integrity and strongly induced cell death at 72 h. This result was supported by Volk and Mundt ([2007]) who studied the cytotoxicity of exometabolites from *Nostoc insulare* culture, resulting in human amniotic epithelial cell death. There are some reports about the effect of cyanobacterial toxins on plant cell death. For example, microcystin-RR from *Microcystis aeruginosa* decreased cell viability of tobacco (*Nicotiana tabacum* L. cv. BY-2) cell line after treatment for 144 h (Yin et al. [2005]). Sanevas et al. ([2007]) reported that crude extract from *Hapalosiphon* sp. caused cell death in onion (*Allium cepa* L. cv. Raputa II) and wheat (*Triticum aestivum* L. cv. Norin 61) roots. In general an increase in the number of cell death when the concentration and time were increased correlated with activation of ROS production, suggesting ROS caused oxidative stress and cell death despite increased antioxidative enzyme activities. This may be due to an imbalance that the production of ROS is over antioxidant defenses.

**Figure 5:**
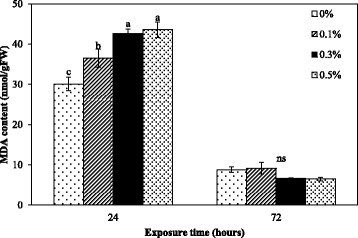
**MDA content of** ***M*** **.** ***pigra*** **roots exposed to different concentrations of the extract for 24 and 72 h.** Data are mean values of three replications and SD are indicated by vertical bars. The different letters above the column indicate significant differences (*p* ≤ 0.05) at each time by DMRT. ns = no significant difference.

Effect of *Nostoc* sp. extract on lipid peroxidation was investigated by determining malondialdehyde (MDA) content, which is a by-product of the peroxidation of membrane lipids. MDA content at 24 h significantly increased with higher concentrations. There was no significant difference between MDA content at various concentrations and the control at 72 h after exposure (Figure 5). The results showed that elevated lipid peroxidation, indicator of oxidative damage, is an important feature of the toxicity of the extract to *M. pigra* roots. These results agree with other cyanobacterial extract that have involved MDA content and lipid peroxidation. It has been reported that microcystins from cyanobacterial bloom material induced oxidative damage, such as lipid peroxidation, in alfalfa (*Medicago sativa* L.) seedlings (Pflugmacher et al. [2006]). Microcystin-LR can increase MDA content in *Vallisneria natans* (Lour.) Hara (Jiang et al. [2011]). Koodkaew et al. ([2012a], [b]) showed that ambiguine D isonitrile and hapalocyclamide from the cyanobacterium, *Hapalosiphon* sp., increased MDA content and caused cell death in lettuce (*Lactuca sativa* L.) seedlings. Normally, MDA levels can be correlated with ROS levels (Yin et al. [2005]; Koodkaew et al. [2012a], [b]). The extract might induce ROS production in the cells and cause oxidative damage such as peroxidation of lipids, causing initial cell death at 24 h. The lack of a difference of MDA content at 72 h might be the result of extremely damaged cell membrane leading to metabolite leakage under longer of exposure because it correlated with the severe cell death at 72 h after treatment (Figure 4).

**Figure 6:**
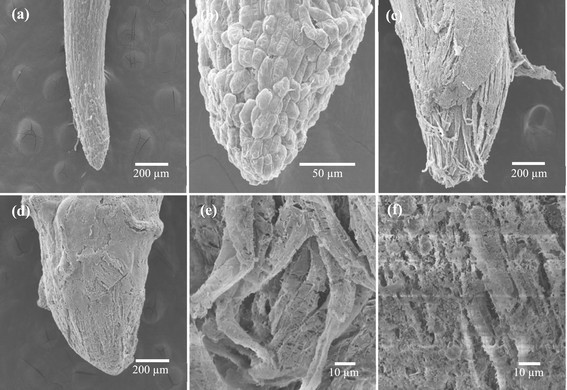
**SEM micrographs of** ***M*** **.** ***pigra*** **root tips treated with an extract from** ***Nostoc*** **sp. for 72 h. (a)** control (magnification = 80x) **(b)** healthy root cap from the control (500x) **(c,** **d)** roots grown in 0.5% of the extract (80x) and **(e,** **f)** root tip cell damage in the treatment with 0.5% of the extract (1,000x).

Changes of root ultrastructure after *Nostoc* sp. extract exposure were investigated by using electron microscope. After 72 h of exposure, SEM micrographs showed completely changed root tip cells/structures between the control and the treated roots. The control roots were narrower and more elongated than those of the treated roots (Figure 6). The root tip cells and epidermal cells of the control roots were intact while the epidermis peeled off and the root cap destructed in the treated roots (Figure 6a-b, c-d). These ultrastructural changes confirmed that cell damage was caused by the extract. It has been reported that some allelochemicals such as two sesquiterpene-derivatives from *Ageratina adenophora* (Asteraceae) caused irregular shapes of root tip cells with a greater number of separated cells and the collapse of the root cap cells (Yang et al. [2011]). Burgos et al. ([2004]) reported that allelochemical from rye (*Secale cereal* L.) reduced the differentiation of root cap cells of cucumber (*Cucumis sativus* L.) seedlings. The extract had severe effects on root cap and epidermal cells. It might be caused by the susceptibility of root cap and epidermal cells to the environment. The extract could easily penetrate the cell walls and enter the cells.

**Figure 7:**
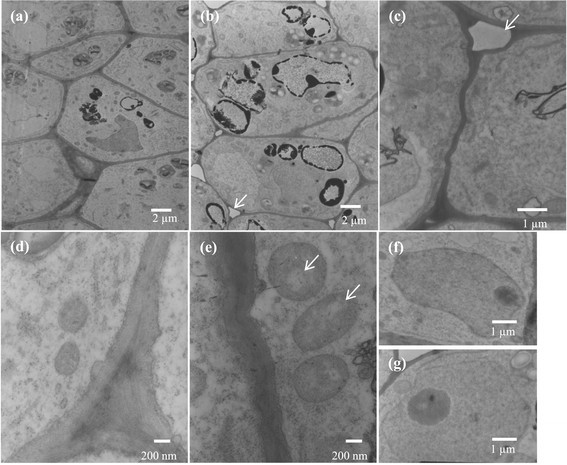
**TEM micrographs of** ***M*** **.** ***pigra*** **root tips treated with an extract from** ***Nostoc*** **sp. for 72 h. (a)** control (magnification = 2,500x) **(b)** treated root (2,500x) showing some substances stored vacuoles and the dissolution of the middle lamella (white arrow) **(c)** treated root (8,000x) showing the dissolution of the middle lamella (white arrow) **(d)** mitochondria of the control (20,000x) **(e)** swollen mitochondria in treated root (20,000x) showing indistinct cristae (white arrows) **(f** **, g)** normal nucleus of control and treated root (5,000x).

Disaggregation and destruction of root tip cells (Figure 6e-f) indicate that the extract affected the cell structure by disintegrating the middle lamella as shown in a TEM micrograph (Figure 7b-c). Beers ([1997]) reported that the dissolution of middle lamella and cell wall occur when plant cells undergo cell death. Roots response to environmental stress by the increase of lignin production resulting cell wall lignification and stunted roots (Siegel [1993]). Disaggregation of root tip cells might be caused by the continued expansion of the stele and a rigidification of the roots. TEM micrographs also showed swollen mitochondria and indistinct cristae in treated roots (Figure 7e). Mitochondria are one of the major sources of ROS in roots (Mano [2002]; Gill and Tuteja [2010]) which can cause oxidative damage in cells. Jones ([2000]) reported that swollen organelles and broken plasma membranes are stages of cell death when metabolic homeostasis fails and mitochondria contribute to apoptotic cell death. Continuous damage of mitochondria becomes irreversible and ensures cell death (Van Loo et al. [2002]). Diaz-Tielas et al. ([2012]) showed that alteration of mitochondrial membranes, swollen mitochondria, and the induction of irreversible cell death were due to the phytotoxicities of chalcone on *Arabidopsis thaliana* (L.) Heynh. seedlings.

## Conclusions

Based on our results, we conclude that a *Nostoc* sp. extract enhanced oxidative stress by ROS production resulting in lipid peroxidation of cell membranes and cell death despite the activation of antioxidative enzymes. This was confirmed by destruction of root tip cells and aberrant mitochondria. These results are consistent with our previous study that the extract inhibited root growth of *M. pigra* by inducing oxidative stress and cell death. To clarify the mechanism of action of *Nostoc* sp. extract provides benefit information for application to use as natural herbicide and control *M. pigra* − invasive weed in some parts of the world. The extract might be developed to use as bioherbicide for weed control. The effects of this extract on other weed species, field experiment, and purification of bioactive compounds need to be investigated.

## Authors’ original submitted files for images

Below are the links to the authors’ original submitted files for images. Authors’ original file for figure 1 Authors’ original file for figure 2 Authors’ original file for figure 3 Authors’ original file for figure 4 Authors’ original file for figure 5 Authors’ original file for figure 6 Authors’ original file for figure 7

## Abbreviations

APX: Ascorbic acid peroxidase
CAT: Catalase
DHE: Dihydroethidium
GR: Glutathione reductase
H_2_O_2_: Hydrogen peroxide
HO^•^: Hydroxyl radical
MDA: Malondialdehyde
NADPH: Nicotinamide adenine dinucleotide phosphate
O_2_•^−^: Superoxide anion radical
PODs: Peroxidases
ROS: Reactive oxygen species
SEM: Scanning electron microscope
TEM: Transmission electron microscope
