# Supplementary figures and images for: Nostoc sp. extract induces oxidative stress-mediated root cell destruction in Mimosa pigra L
Source: Bot Stud. 2015 Feb 22;56:3. doi: 10.1186/s40529-014-0081-3 (PMC5432888; doi:10.1186/s40529-014-0081-3)

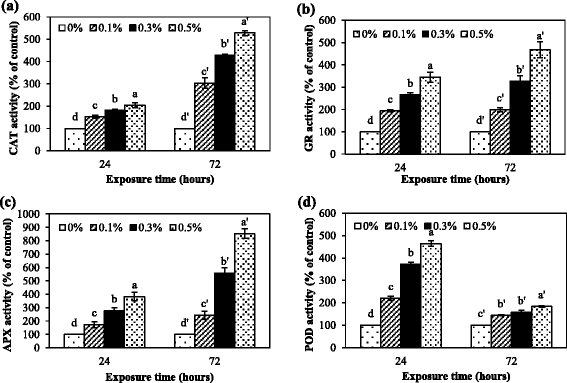

Supplement: Supplementary file 1 — Authors’ original file for figure 1 [file 40529_2014_81_MOESM1_ESM.gif]

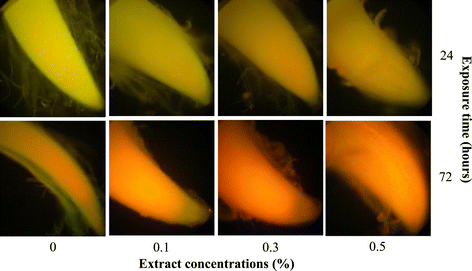

Supplement: Supplementary file 2 — Authors’ original file for figure 2 [file 40529_2014_81_MOESM2_ESM.gif]

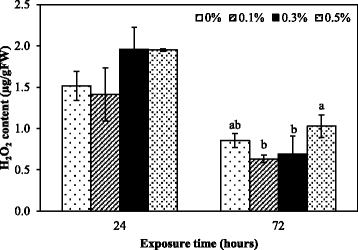

Supplement: Supplementary file 3 — Authors’ original file for figure 3 [file 40529_2014_81_MOESM3_ESM.gif]

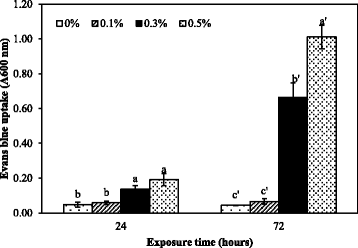

Supplement: Supplementary file 4 — Authors’ original file for figure 4 [file 40529_2014_81_MOESM4_ESM.gif]

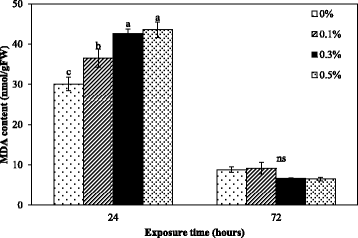

Supplement: Supplementary file 5 — Authors’ original file for figure 5 [file 40529_2014_81_MOESM5_ESM.gif]

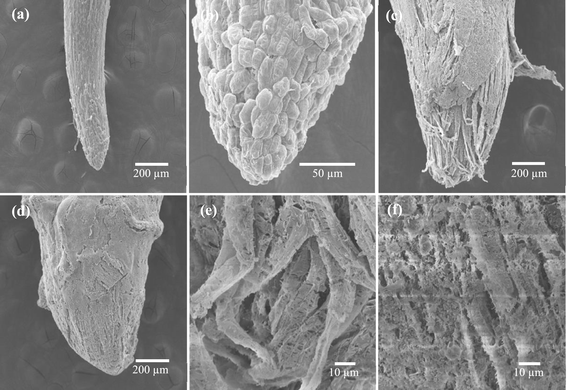

Supplement: Supplementary file 6 — Authors’ original file for figure 6 [file 40529_2014_81_MOESM6_ESM.gif]

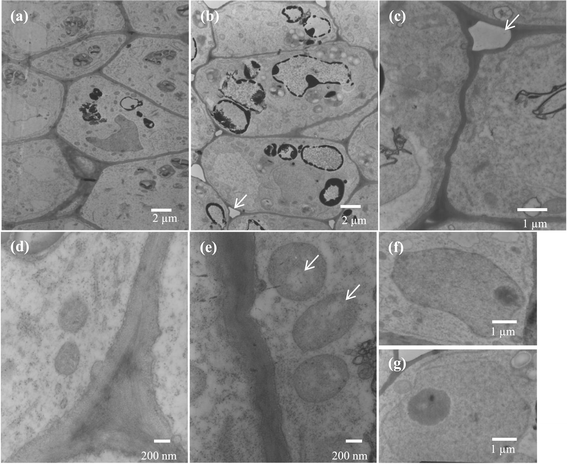

Supplement: Supplementary file 7 — Authors’ original file for figure 7 [file 40529_2014_81_MOESM7_ESM.gif]
